# Supplementary material for: Oscillatory cortical forces promote three dimensional cell intercalations that shape the murine mandibular arch
Source: Nat Commun. 2019 Apr 12;10:1703. doi: 10.1038/s41467-019-09540-z (PMC6461694; doi:10.1038/s41467-019-09540-z)
Supplement: Supplementary file 1 — Supplementary information [file 41467_2019_9540_MOESM1_ESM.pdf]

Supplementary Figure 1

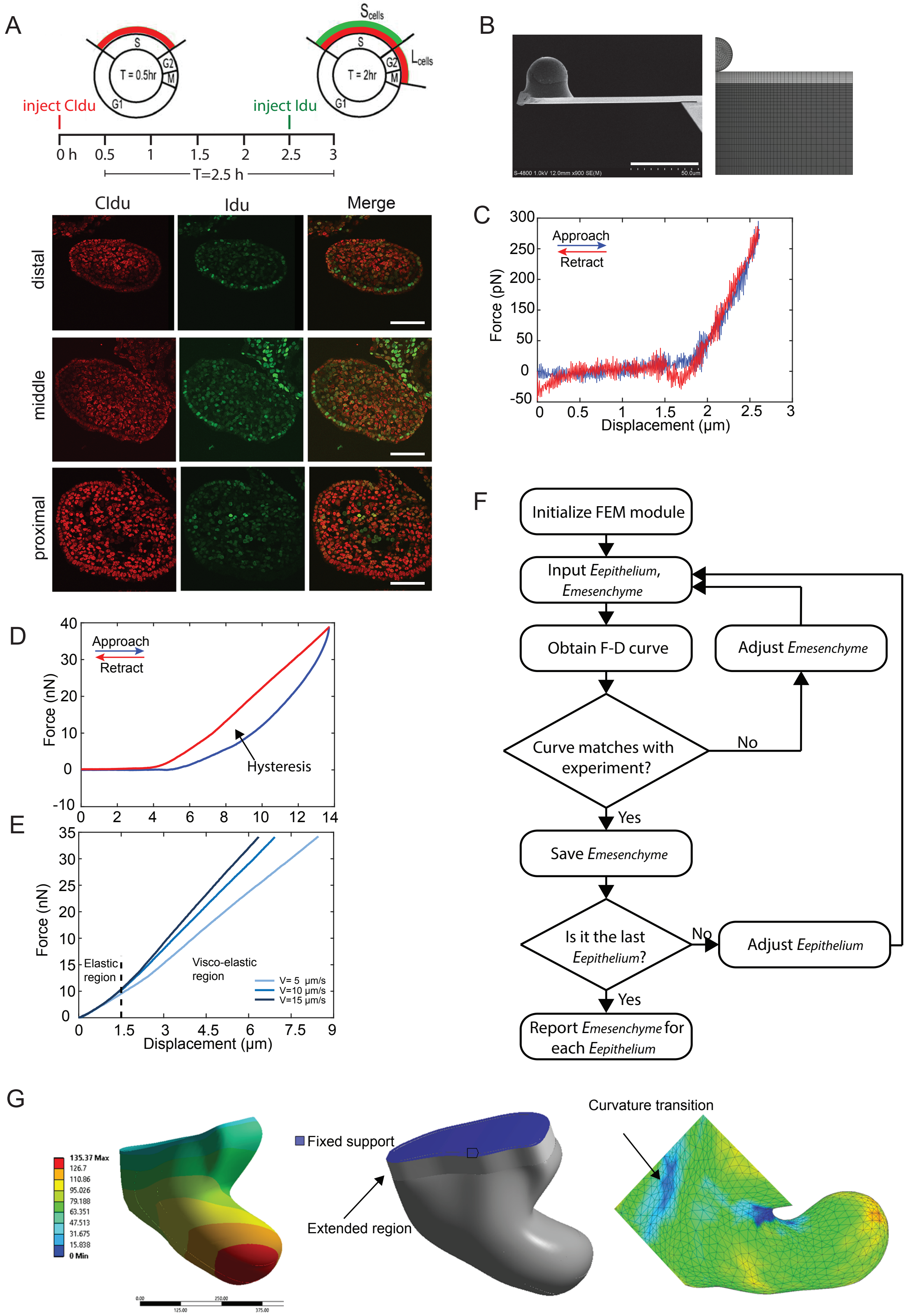

Supplementary Figure 1

Viscoelastic properties of the mandibular arch. A Method of cell cycle time measurement using dual thymidine analog labelling to mark cells in S phase. The proportion of CIdu- and Idu-positive cells were quantified after immunostaining to calculate cell cycle time in different regions of the mandibular arch. Scale bar: 80 µm. **B** Scanning electron micrograph of an AFM cantilever with a 30 µm spherical tip employed for embryo indentation (left) and the axisymmetric mesh of the finite element model (right). Scale bar: 50 µm. **C** A representative force-displacement (F-D) curve of small indentation is consistent with purely elastic behaviour. **D** A representative force-displacement curve under large indentation reveals evidence of hysteresis, or energy dissipation that indicates viscoelastic behaviour. **E** Force-displacement curves at different indentation rates indicate viscoelastic behaviour. **F** Flowchart of finite element model (FEM) simulation for determining the elastic modulus of mesenchyme. **G** Finite element-predicted deformation (in µm) (left); boundary condition set for simulation (centre); curvature transition used for determining the proximal end (right).

Supplementary Figure 2

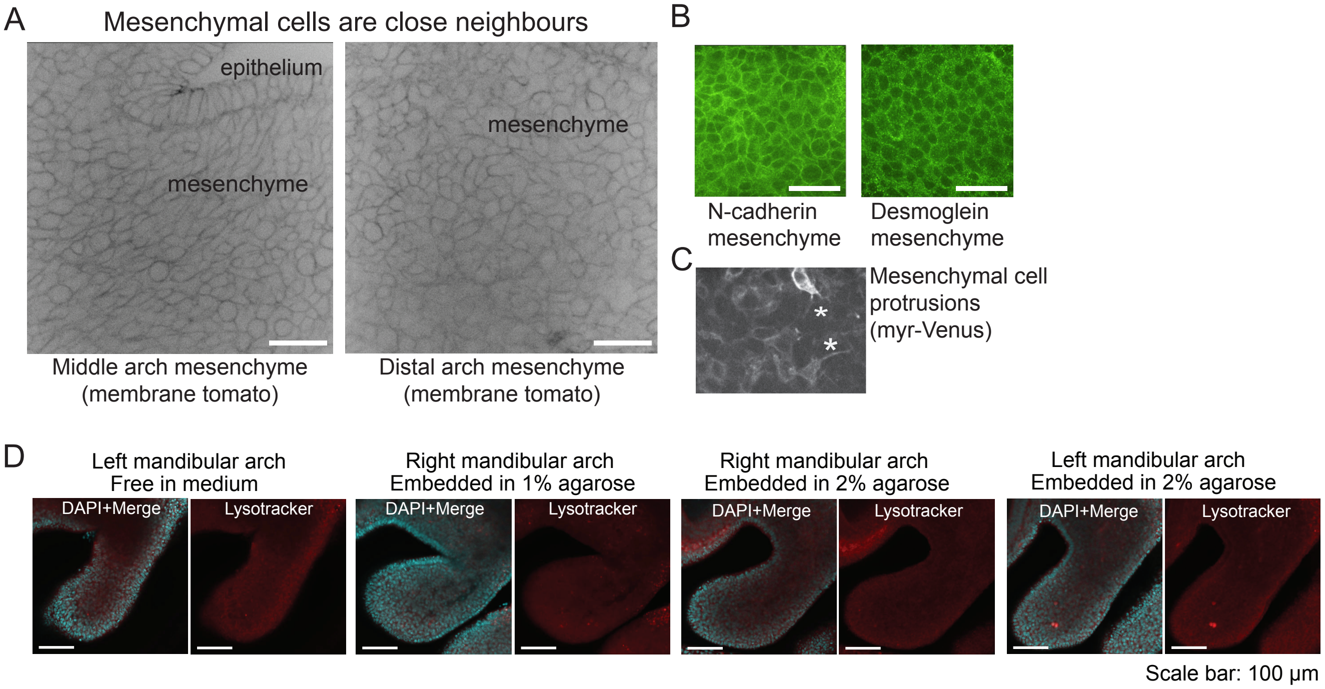

E

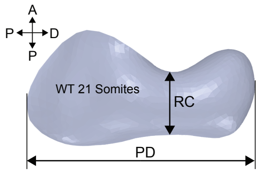

RC/PD	Uncultured WT	Free in medium (WT 20 somites)	1% agarose (WT 20 somites)	1% agarose without rat serum (WT 20 somites)	2% agarose (WT 20 somites)
Start	0.413 (19 somites)	0.373	0.354	0.357	0.379
End	0.296 (21 somites)	0.327	0.313	0.321	0.356
Shape change	28.264%	12.205%	11.414%	9.934%	6.039%

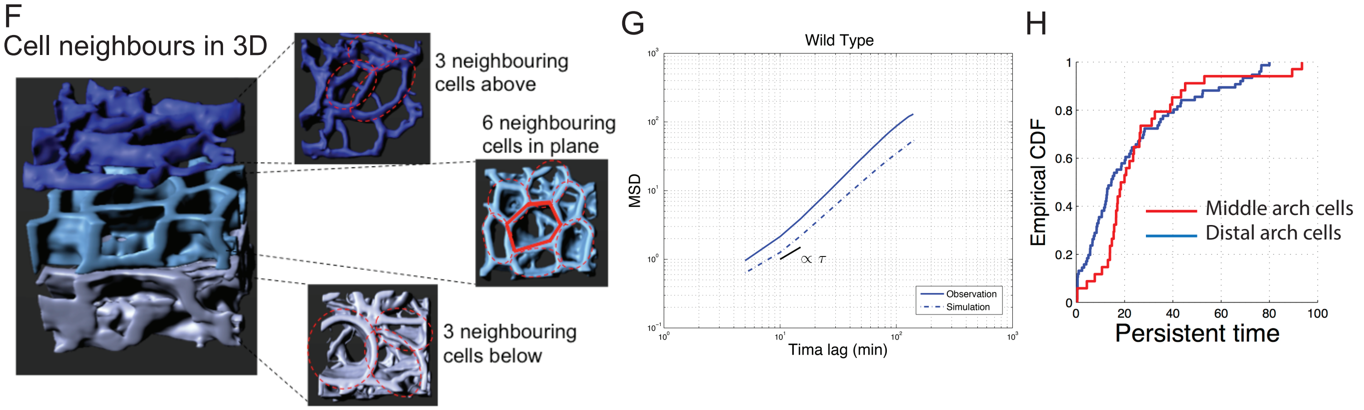

Random walk-simulated cell trajectories

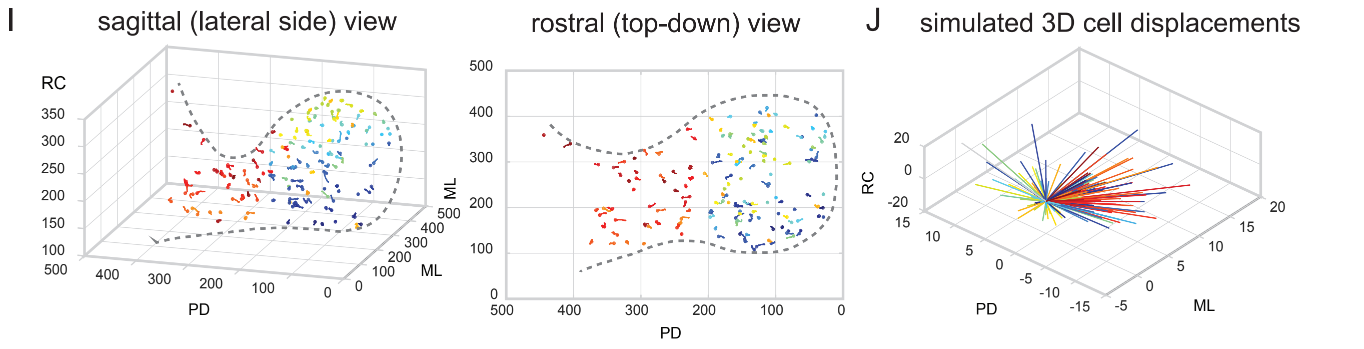

Supplementary Figure 2
Mesenchymal cell neighbour relationships and random walk model. A Mesenchymal cells in live *mTmG* embryos are intimate neighbours without visible intercellular spaces by confocal microscopy (representative of minimun 5 embryos at 20 somite stage). **B** Mesenchymal cells express abundant cell-cell adhesion proteins N-cadherin (left) and desmoglein (right) (representative of 2 embryos each at 20-21 somite stage). **C** Mosaic expression of *myr-Venus* among mesenchymal cells in the arch revealed protrusive activity that may facilitate cell rearrangements (representative of 2 embryos at 20 somite stage). **D** Live embryos were embedded in a cylinder of agarose used for light sheet imaging. Culture in nil, 1% and 2% agarose made little difference in detectable apoptosis as assessed by Lysotracker staining. **E** Change in the width/length ratio (rostrocaudal/proximodistal RC/PD) of the mandibular arch was slow during 4 h in culture compared to development *in utero*, and worse in 2% agarose. **F** Cell neighbours in 3D were counted by examining small volumes of membrane segmented mesenchymal tissue among live *mTmG* embryos. **G** According to a random walk model that we employed (methods), mean squared displacement (MSD), a measure of the extent of random motion, was similar between observed and simulated cell trajectories, thereby validating this model for statistical comparison between different regions of the arch. **H** Cells in the middle waist region (red curve) exhibited a greater slope for cumulative distribution function (CDF) of persistent time compared to those in the distal bulbous region (blue curve, $p<0.01$), implying motion of waist cells is more consistent over time compared to those of the bulbous region. **I, J** Simulated cell trajectories based on our random walk model (compare with Fig. 2F, G).

Supplementary Figure 3

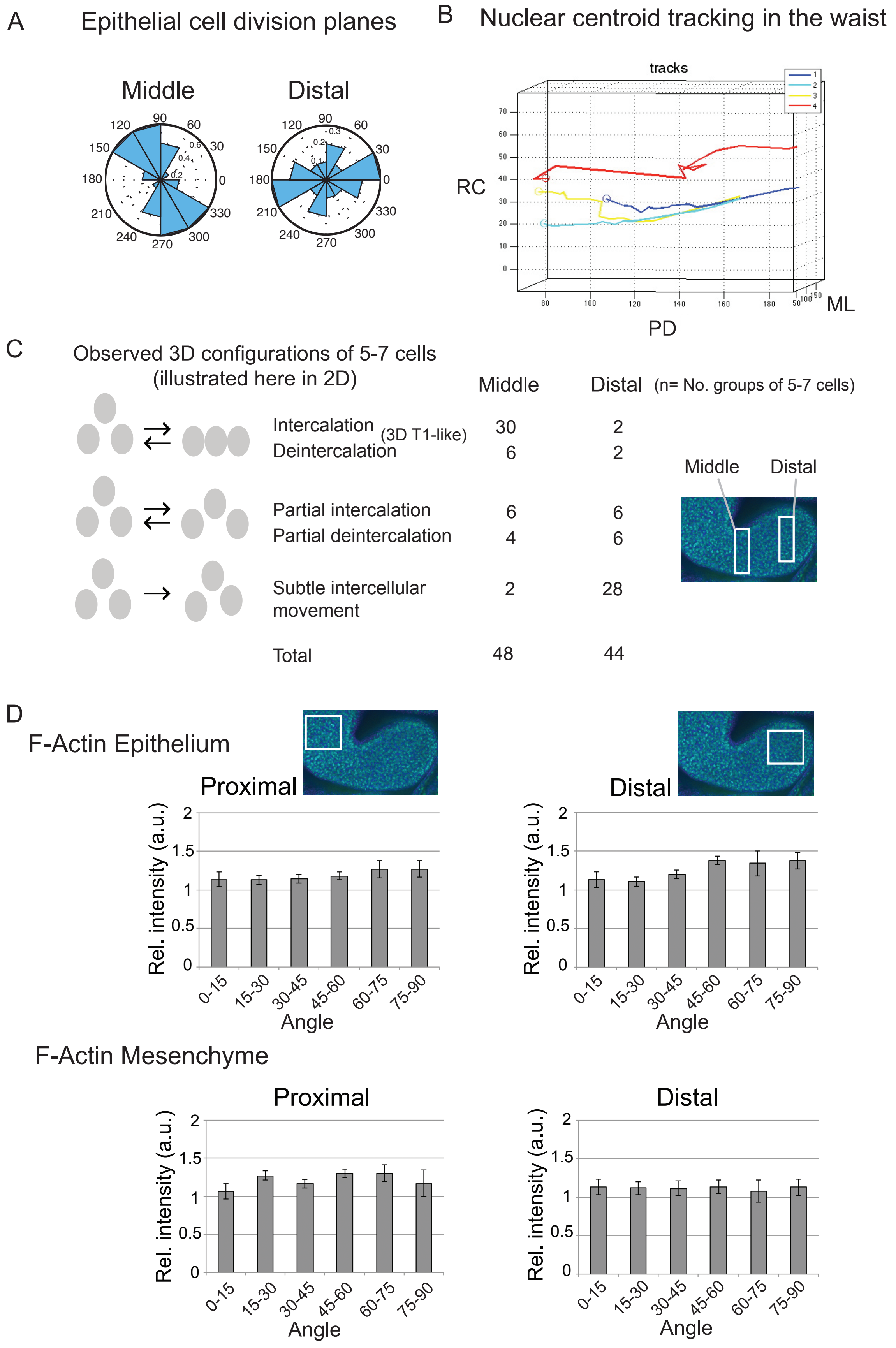

Supplementary Figure 3
Proportion of three dimensional mesenchymal cell neighbour interactions.
A Epithelial cell division planes in the middle and distal regions of the mandibular arch. **B** Tracks of neighbouring mesenchymal nuclei reveal AP convergence of cell tracks during proximodistal elongation of the middle region. **C** Proportion of different dynamic configurations of 5-7 mesenchymal cells that we defined in the middle and distal region. **D** The angular distribution of immunostain fluorescence intensity for epithelial (n = 4 embryos) and mesenchymal (n = 4 embryos) F-actin in proximal and distal regions of the arch relative to the arch long axis that was designated as 0° was quantified using SIESTA. Asterisks denote $p<0.05$, Student's *t*-test, error bars denote s.e.m. Source data are provided as a Source Data file.

Supplementary Figure 4

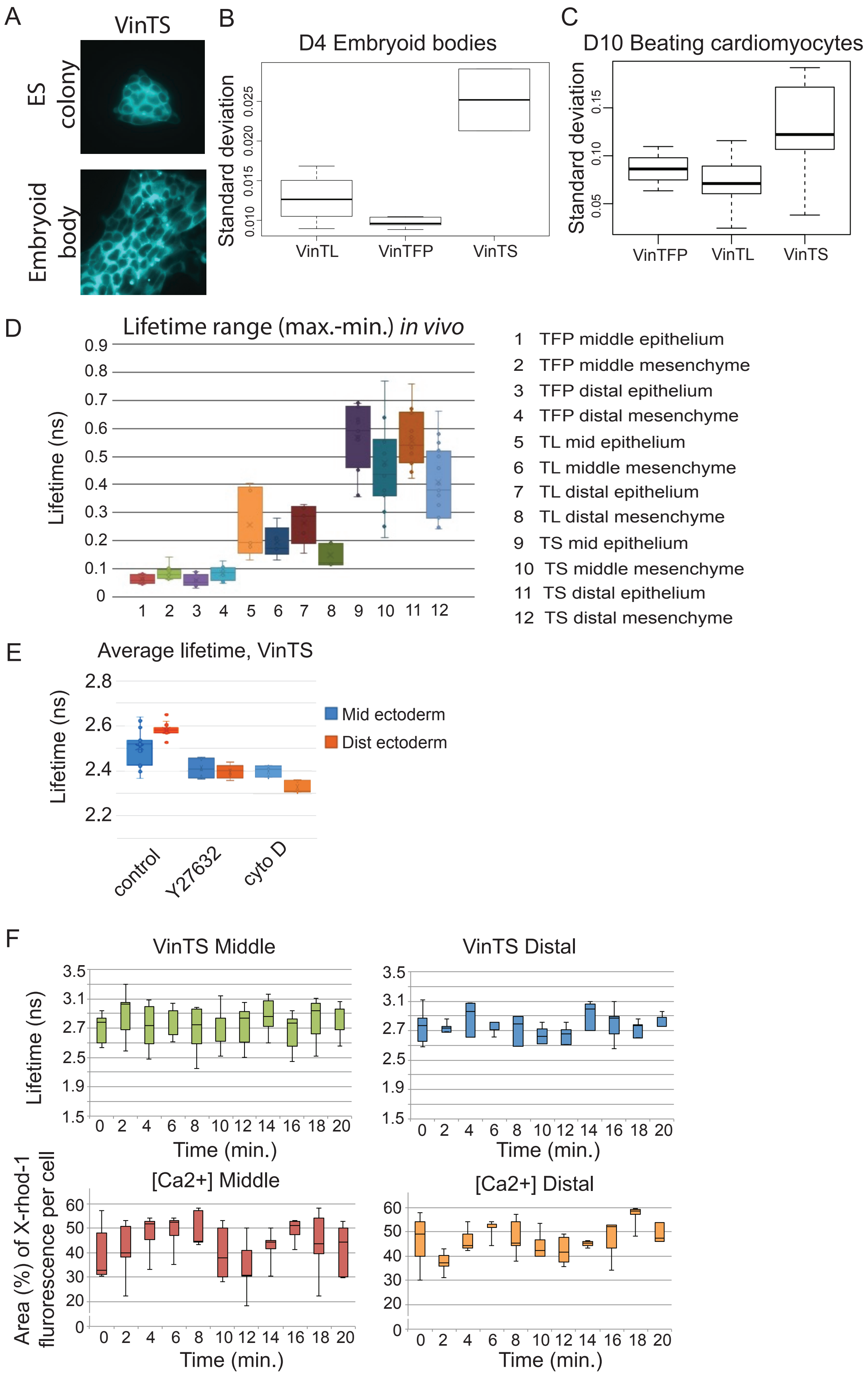

Supplementary Figure 4

Vinculin force sensor evaluation *in vitro* and *in vivo*. A Expression of the full length tension sensor knock-in VinTS in an ES cell colony and an embryoid body (representative of minimum 20 colonies and embryoid bodies). **B, C** Standard deviation of full length vinculin tension sensor (VinTS), TFP (FRET donor) only control (VinTFP), and vinculin tailless control (VinTL) in embryoid body cells (B) and differentiated beating cardiomyocytes (C) (n = 2-11 colonies per condition). **D** Dynamic range of lifetimes values observed in middle (mid) and distal (dist) epithelium and mesenchyme of the 20 somite stage mandibular arch for each of the VinTFP, VinTL and VinTS mouse lines, n = 10 interval time lapse assessments per 5-15 cells in each of 2-3 embryos, error bars indicate standard deviation. **E** VinTS lifetime in the mid-portion of the arch was dampened by treatment of embryos with Y27632, a ROCK inhibitor (E) and cytochalasin D, an actin polymerisation inhibitor (F) (n = 15 cells per region in each of 3-4 embryos, error bars denote s.e.m; compare with 4C). **F** VinTS fluorescence lifetime and X-rhod-1 fluctuation data that correspond to correlations in Fig. 4F. Area % refers to the proportion of X-rhod-1 fluorescence per cell outlined by VinTS in a single confocal plane quantified using Image J (18 cells per region). Source data are provided as a Source Data file.

Supplementary Figure 5

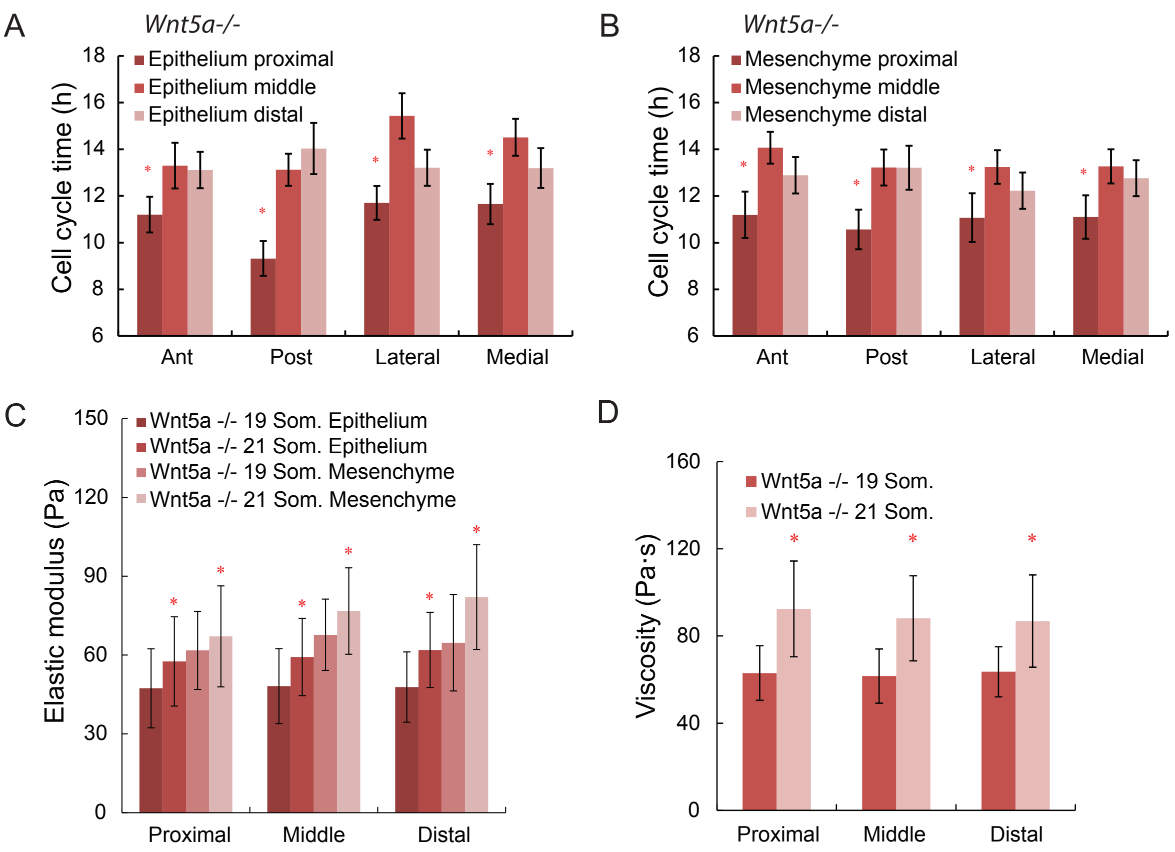

E

Observed 3D configurations of 5-7 cells (illustrated here in 2D)

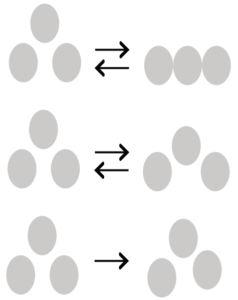

	Wnt5a-/- Middle	*Wnt5a-/-* Distal (n= No. groups of 5-7 cells)
Intercalation (3D T1-like)	5	3
Deintercalation	4	2
Partial intercalation	8	4
Partial deintercalation	5	6
Subtle intercellular movement	16	22
Total	38	37

Middle Distal

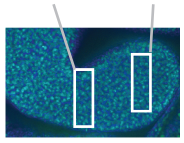

Supplementary Figure 5

Cell cycle times, viscoelastic properties, and mesenchymal cell rearrangements in the *Wnt5a*$^{-/-}$ mutant mandibular arch. A, B Epithelial (A) and mesenchymal (B) cell cycle times in in 24 adjacent regions of the mandibular arch (as in Fig. 1B). As in WT embryos (see Fig. 1C, D), cell division was more rapid in the proximal region for both epithelial and mesenchymal layers; n = 3 embryos at 20 somite stage, 15-35 cells examined for each of 12 epithelial regions per embryo, 50-75 cells examined for each of 12 mesenchymal regions per embryo; asterisks denote $p<0.05$, Student's *t*-test. **C** Elastic (Young's) modulus (stiffness) of epithelium and mesenchyme. **D** Viscosity of whole tissue in proximal, middle and distal regions of the mandibular arch at 19 and 21 somite stages. In contrast to WT embryos (see Fig. 1F), middle arch epithelial stiffness did not increase as significantly between 19 and 21 somite stages. For C and D, 15 separate sites in each proximal, middle and distal region were indented in triplicate (45 measurements per region) per embryo; n = 3 embryos. Asterisks denote statistical significance, p value range: $0.02\text{-}10^{-7}$, two-tailed *t*-test error bars denote standard deviation. **E** Proportion of dynamic configurations of 5-7 cells in the middle and distal regions of the *Wnt5a*$^{-/-}$ mutant mandibular arch. Source data are provided as a Source Data file.

Supplementary Figure 6

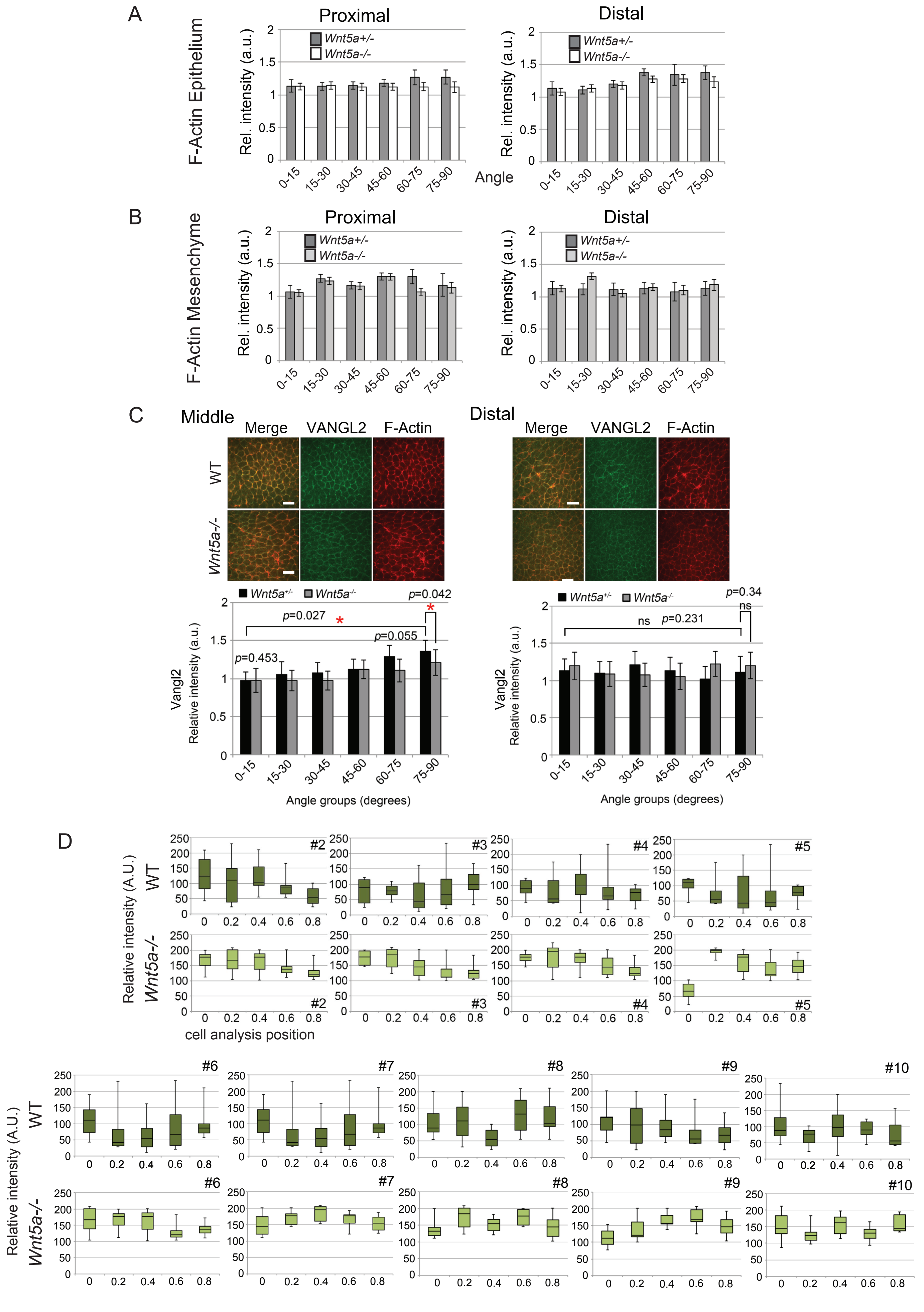

Supplementary Figure 6
Cortical polarity is not biased in proximal and distal regions of the mandibular arch. A, B F-actin orientation in proximal and distal regions for epithelium (A) and mesenchyme (B) in WT and *Wnt5a*$^{-/-}$ mutant embryos that corresponds to Fig. 6A. **C** VANGL2 immonostain intensity was biased parallel to actomyosin along proximodistal cell interfaces in the WT middle, but not distal, arch. Scale bars: 20 µm. That bias was diminished in *Wnt5a*$^{-/-}$ mutants. **D** Variation of cytosolic calcium concentration in individual cells over time; corresponds to Fig. 6E. Source data are provided as a Source Data file.

Supplementary Figure 7

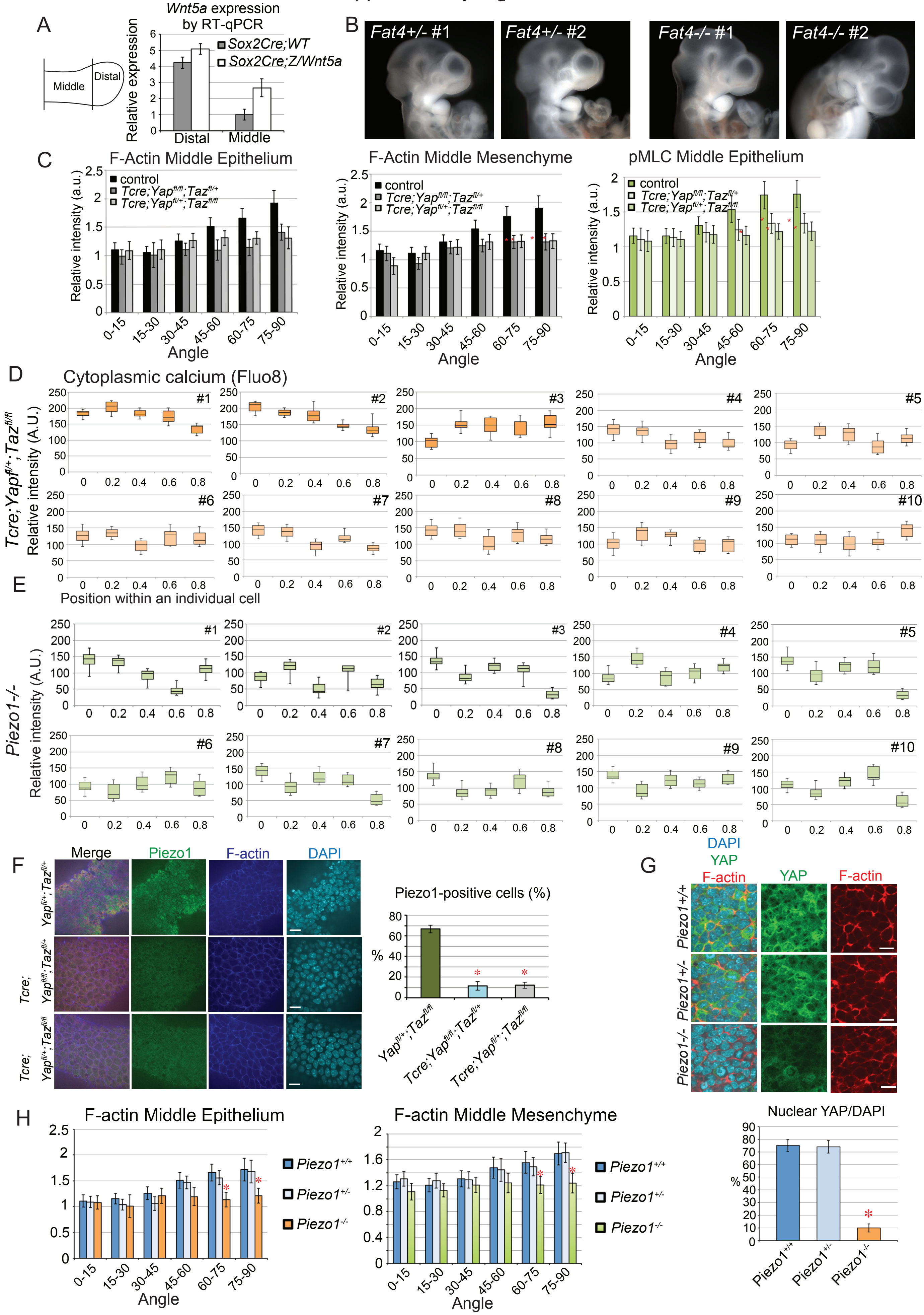

Supplementary Figure 7

Crosstalk between *Yap/Taz* and *Piezo1*. A Quantitative RT-PCR of middle and distal branchial arch sections dissected from WT or *Sox:Cre;Z/Wnt5a* embryos. **B** *Fat4*$^{-/-}$ embryos at 20-21 somite stage exhibit morphologically normal branchial arches. **C** F-actin and pMLC biases were diminished in 20-21 somite *T:Cre;Yap*$^{f/f}$*;Taz*$^{f/+}$ and *T:Cre;Yap*$^{f/+}$*;Taz*$^{f/f}$ embryos. Asterisks indicate $p<0.05$, Student's *t*-test. **D** Cytosolic calcium fluctuations in the 20-21 somite *T:Cre;Yap*$^{f/+}$*;Taz*$^{f/f}$ arch was diminished relative to WT embryos. Each graph represents one cell. **E** Cytosolic calcium fluctuations in the 20-21 somite *Piezo1*$^{-/-}$ arch was diminished relative to WT embryos. **F** PIEZO1 immunostain intensity was diminished in the absence of any three *Yap/Taz* alleles at the 20-21 somite stage (scale bar 10 μm). **G** Proportion of cells exhibiting nuclear YAP in the 20-21 somite *Piezo1*$^{-/-}$ middle mandibular arch (scale bar 10 μm). **H** Rostrocaudal F-actin biases were diminished in 20-21 somite *Piezo1*$^{-/-}$ embryos. Source data are provided as a Source Data file.

Supplementary Table 1

Parameters incorporated into the finite element model:

		Elastic modulus (Pa)			Viscosity (Pa·s)		
		Proximal	Middle	Distal	Proximal	Middle	Distal
WT	Epithelium	42.31→71.17	46.83→109.17	48.42→73.71	77.98→127.53	68.18→136.38	71.33→126.84
	Mesenchyme	55.53→127.90	61.10→107.62	68.12→130.25			
Wnt5a -/-	Epithelium	47.36→57.58	48.20→59.30	47.83→61.96	62.97→92.39	61.62→88.13	63.58→86.81
	Mesenchyme	61.79→67.11	67.73→76.78	64.69→82.11			

		Cell cycle time (hrs)											
		Proximal				Middle				Distal			
		Ant.	Post.	Dorsal	Ventral	Ant.	Post.	Dorsal	Ventral	Ant.	Post.	Dorsal	Ventral
WT	Epithelium	11.12	9.23	11.22	11.45	15.3	13.65	14.21	14.25	14.1	13.98	14.23	14.21
	Mesenchyme	10.21	10.22	10.34	10.44	13.21	12.22	13.33	13.34	12.22	11.31	12.67	12.71
Wnt5a -/-	Epithelium	11.2	9.32	11.7	11.65	13.3	13.12	15.43	14.51	13.11	14.03	13.21	13.19
	Mesenchyme	11.19	10.57	11.07	11.1	14.07	13.22	13.24	13.27	12.89	13.21	12.23	12.76
