# Supplementary material for: Oscillatory cortical forces promote three dimensional cell intercalations that shape the murine mandibular arch
Source: Nat Commun. 2019 Apr 12;10:1703. doi: 10.1038/s41467-019-09540-z (PMC6461694; doi:10.1038/s41467-019-09540-z)
Supplement: Supplementary file 3 — Description of Additional Supplementary Files [file 41467_2019_9540_MOESM3_ESM.pdf]

Movie 1
Rendering of 3D OPT image of the right mandibular arch of a 19 somite stage WT embryo.

Movie 2
Rendering of 3D OPT image of the right mandibular arch of a 21 somite stage WT embryo.

Movie 3
Rendering of 3D OPT image of the predicted shape of the right mandibular arch of a 21 somite embryo based on our finite element model. Four hours of growth (between 19-21 somite stages) were simulated starting with the shape of the actual WT 19 somite stage arch. Inputs included spatial distributions of cell cycle time, epithelial and mesenchymal Young's modulus, and whole tissue viscosity.

Movie 4
Three dimensional rendering of the right mandibular arch of a 20 somite *CAG::H2B-GFP* embryo imaged by live light sheet microscopy. Nuclei in the narrow mid-portion were oriented with their long axes perpendicular to the distalward axis of growth.

Movie 5
Cell membranes have been segmented in this 3D rendering of the surface epithelium together with a partial volume of the underlying mesenchyme in a 20 somite embryo harbouring the *mTmG* transgene for membranes and *CAG::H2B-GFP* for nuclei. Live light sheet microscopy of the intact embryo was performed with 10 min. time lapse intervals. Distal is to the lower left.

Movie 6
Cells surrounding one mesenchymal cell (blue) have been coloured to facilitate counting the number of adjacent neighbours.

Movie 7
Three dimensional trajectories of a subset of nuclei labelled with H2B-GFP in a 20 somite WT embryo.

Movie 8
Three dimensional start point-to-end point displacements (dandelion plot) of cells labelled in Mov. 7 with colour code corresponding to Fig. 2G. Red/orange cells representing the middle region move predominantly distalward whereas green/blue cells in the distal region move radially.

Movie 9
Simulation of cell tracks that was based on our random walk model and was started from the initial frame of the same time lapse experiment shown in Mov. 7.

Movie 10
Dandelion plot derived from random walk simulation shown in Mov. 9.

Movie 11
Proximodistal-rostrocaudal plane taken from a light sheet movie of the 20 somite stage mandibular arch with 10 min. time lapse intervals over 150 min.. The superimposed strain

grid is based on nodes that remain fixed to the same positions throughout the movie and highlight tissue deformation over time.

Movie 12
Sagittal view of an entire mandibular arch of a 20 som. *CAG::H2B-GFP*. Distal is to the right, and time lapse intervals were 10 min. Convergence of cells in the mid-portion is suggested by their centripetal movements.

Movie 13
An intermediate-scale volume of tissue of the middle arch of a 20 somite *CAG::H2B-GFP* embryo. Distal is to the right. The movie begins with an approximately rostral (top-down) view then turns to stabilise on a sagittal (lateral) view for the majority of the movie before returning back to a rostral view at the end. Time lapse intervals were 10 min..

Movie 14
A small-scale volume of several cells from within the middle region of the mandibular arch of a 20 somite *CAG::H2B-GFP* embryo. The predominant view is sagittal, and distal is to the right. One central nucleus intercalated among several others in the direction of its long axis that is perpendicular to the axis of tissue growth. Time lapse intervals were 10 min..

Movie 15
Another example of a small-scale volume of several cells from within the middle region of the mandibular arch of a 20 somite *CAG::H2B-GFP* embryo. This movie rotates view about the rostrocaudal axis. One central nucleus intercalated among several others in the direction of its long axis that is perpendicular to the axis of growth. Time lapse intervals were 10 min..

Movie 16
Combined H2B-GFP and mTmG labels with membrane rendering (red) shows one cell (arrow at the beginning) intercalate and separate neighbours caudal to (below) it within the middle region of the right mandibular arch of a 20 somite WT embryo. The view is sagittal-rostral oblique to best show the intercalation event. Time lapse intervals were 10 min..

Movie 17
A small-scale volume of several cells from within the distal portion of the mandibular arch of a 20 somite *CAG::H2B-GFP* embryo. The movie begins and ends with rostral views but the predominant view is from a distal perspective. Nuclei subtly adjusted their positions relative to one another but did not intercalate. Time lapse intervals were 10 min..

Movie 18
Live, colour-coded fluorescence lifetime evaluation of vinculin tension sensor (VinTS) among epithelial cells in the mandibular arch of an intact mouse embryo visualised by confocal microscopy. Time lapse intervals were 2 min..

Movie 19
Cytosolic fluorescence intensity of the X-rhod-1 calcium indicator fluctuated asynchronously among cells in the middle region of the WT mandibular arch (see Fig. 4F, Supplementary Fig. 4G for quantification). Time lapse intervals were 2 min..

Movie 20
X-rhod-1 calcium indicator fluctuation among cells in the distal region of the WT mandibular arch (see Fig. 4F, Supplementary Fig. 4G for quantification). Time lapse intervals were 2 min..

Movie 21
Rendering of 3D OPT image of the right mandibular arch of a 19 somite stage *Wnt5a*$^{-/-}$ mutant.

Movie 22
Rendering of 3D OPT image of the right mandibular arch of a 21 somite stage *Wnt5a*$^{-/-}$ mutant.

Movie 23
Rendering of 3D OPT image of the predicted shape of the right mandibular arch of a 21 somite *Wnt5a*$^{-/-}$ mutant based on our finite element model. Four hours of growth (between 19-21 somite stages) were simulated starting with the actual shape of the 19 somite stage arch of a *Wnt5a*$^{-/-}$ mutant. Inputs included spatial distributions of cell cycle time, epithelial and mesenchymal Young's modulus, and whole tissue viscosity.

Movie 24
Three dimensional trajectories of a subset of nuclei labelled in a 20 somite *CAG::H2B-GFP;Wnt5a*$^{-/-}$ embryo.

Movie 25
Three dimensional start point-to-end point displacements (dandelion plot) of cells labelled in Mov. 24 with colour code corresponding to Fig. 5I. Red/orange cells representing the middle region move radially compared to their WT counterparts.

Movie 26
A small-scale volume of several cells from within the middle region of the mandibular arch of a 20 somite *CAG::H2B-GFP;Wnt5a*$^{-/-}$ embryo. The predominant view is sagittal and distal is down and to the right. Nuclei subtly adjusted positions relative to one another but did not intercalate. Time lapse intervals were 10 min..

Movie 27
Cytosolic fluorescence intensity of the Fluo-8 calcium indicator fluctuated among cells in the middle region of the WT mandibular arch (see Fig. 4F, Supplementary Fig. 4F for quantification). Time lapse intervals were 2 min..

Movie 28
Fluctuation of the cytosolic fluorescence intensity of the Fluo-8 calcium indicator was dampened among cells in the middle region of the *Wnt5a*$^{-/-}$ mutant mandibular arch (see Fig. 6E, Supplementary Fig. 6D for quantification). Time lapse intervals were 2 min..

Movie 29
Rendering of 3D OPT image of the right mandibular arch of a 21 somite stage transgenic *Sox9:Cre;Z/Wnt5a* embryo that ubiquitously overexpressed *Wnt5a*.

Movie 30
Mesenchymal cell movements in a 21 somite *T:Cre;Yap*$^{f/+}$*;Taz*$^{f/f}$ mutant mandibular arch. Intercellular movements are abundant but disoriented and not purely centripetal. Rostral (anterior) is toward to the top and distal is toward the right. Time lapse intervals were 10 min..

Movie 31
Fluctuation of the cytosolic fluorescence intensity of the Fluo-8 calcium indicator was diminished among cells in the middle region of the *Piezo1*$^{-/-}$ mutant mandibular arch (see Supplementary Fig. 7A for quantification). Time lapse intervals were 2 min..

Movie 32
Rendering of 3D OPT image of the right mandibular arch of a 21 somite stage *Piezo1*$^{-/-}$ mutant.
